# Supplementary material for: Phylogenetic diversity of plants alters the effect of species richness on invertebrate herbivory
Source: PeerJ. 2013 Jun 25;1:e93. doi: 10.7717/peerj.93 (PMC3698468; doi:10.7717/peerj.93)
Supplement: Table S1 [file peerj-01-93-s001.doc]

**SUPPORTING INFORMATION**

**Table S1.** **Plant community herbivory, species richness, phylogenetic diversity, and composition for 38 old field plots.** Species composition lists the species present and how many of the four quadrants it was found in (after the colon). Species codes: ACMI *= Achillea millefolium;* APAN *= Apocynum androsaemifolium;* ASSY *= Asclepias syriaca;* CIAR *= Cirsium arvense;* CIVU *= Cirsium vulgare;* COAR *= Convolvulus arvensis;* CORU *= Cornus rugosa;* COSE *= Cornus sericea;* DACA *= Daucus carota;* ERCA *= Erigeron canadensis;* ERPU *= Erigeron pulchelus;* EUCY *= Euphorbia cyparissias;* EUGR *= Euthamia graminifolia;* EUMA *= Eupatorium maculatum;* FRVI *= Fragaria virginiana;* HYPE *= Hypericum perforatum;* LEVU *= Leucanthemum vulgare;* LIVU *= Linaria vulgaris;* LYCI *= Lysimachia ciliata;* MEAL *= Melilotus alba;* MEAR *= Mentha arvensis;* MELU *= Medicago lupulina;* MEPI *= Mentha piperita;* MESA *= Medicago sativa;* MOFI *= Monarda fistulosa;* NECA *= Nepeta cataria;* OEBI *= Oenothera biennis;* PAQU *= Parthenocissus quinquefolia;* PHHE *= Physalis heterophylla;* PLLA *= Plantago lanceolata;* PLMA *= Plantago major;* PONO *= Potentilla norvegica;* POSI *= Potentilla simplex;* POTR *= Populus tremuloides;* PRVU *= Prunella vulgaris;* RHTY *= Rhus typhina;* RULE *= Rubus leucodermis;* RUSE *= Rudbeckia serotina;* SILA *= Silene latifolia;* SOAS *= Sonchus asper;* SOCA *= Solidago canadensis;* SONE *= Solidago nemoralis;* SYCO *= Symphyotrichum cordifolium;* SYER *= Symphyotrichum ericoides;* SYLA *= Symphyotrichum lanceolatum;* SYLT *= Symphyotrichum lateriflorum;* SYNO *= Symphyotrichum novae-angliae;* SYPU *= Symphyotrichum puniceum;* TAOF *= Taraxacum officianale;* THOC *= Thuja occidentalis;* VICR *= Vicia cracca;* VIRA *= Viburnum rafinesquianum;* VIRI *= Vitis riparia*

**Plot Phylogenetic Species Rich- Estimated Pro- Species Composition**

**Diversity (PSE) ness (SR) portional Leaf**

**Damage**

1 0.650 6 0.424 VICR: 4, EUGR: 4, SOCA:4, SYNO: 3, ASSY:3, MOFI: 1

2 0.806 4 0.404 VICR: 4, SOCA: 1, ERCA: 1, LIVU: 4

3 0.815 4 0.542 MESA:4, VICR:2, ASSY:3, LIVU: 2

4 0.815 6 0.393 EUCY: 2, VICR: 2, POSI: 1, RUSE: 1, SOCA: 3, PLLA: 1

5 0.661 6 0.468 MESA: 2, VICR: 2, CIAR: 4, TAOF: 2, SOCA: 4, ASSY: 1

6 0.516 17 0.879 VICR: 2, FRVI: 3, DACA:4, CIAR: 1, EUMA: 1, RUSE: 1, LEVU: 3, ACMI: 3, EUGR: 3, SOCA: 4, SYNO: 3, SYLT: 3, SYLA: 3, ERPU: 1, PRVU: 2, PLMA: 2, PLLA: 1

7 0.676 18 0.773 HUPE: 3, MELU: 1, MEAL: 2, VICR: 3, RULE: 1, DACA: 4,

TAOF: 2, RUSE: 4, LEVU: 4, EUGR: 2, SOCA:4 SYNO: 2,

SYLT: 2, ERPU: 2, MOFI: 4, PRVU: 1, PLMA: 2, PLLA: 4

8 0.392 5 0.672 VICR: 1, CIAR: 3, TAOF: 1, SOCA: 3, SYNO: 2

9 0.724 5 0.357 VICR: 4, CIAR: 3, EUGR: 1, SOCA: 4, ASSY: 2

10 0.496 12 0.782 VICR: 4, DACA: 2, TAOF: 1, EUMA:2, RUSE: 2, ACMI:3,

EUGR: 2, SOCA: 4, SYNO: 4, SYLT: 4, APAN: 2, COSE: 1

11 0.799 7 0.596 MESA: 1, VICR:2, PAQU: 1, CIAR: 1, RUSE: 1, LEVU: 1,

COAR: 4

12 0.634 4 0.753 VICR: 2, CIAR: 4, TAOF: 4, ASSY: 1

13 0.788 5 0.725 VICR: 3, RUSE: 2, SOCA: 3, ASSY: 1, COAR: 4

14 0.826 6 0.372 MESA: 1, VICR: 2, VIRI: 1, CIAR: 3, TAOF: 1, ASSY: 2

15 0.466 11 0.644 VICR: 4, DACA:2, CIAR: 1, TAOF: 3, RUSE: 1, LEVU: 2,

EUGR: 2, SOCA: 4, SYNO: 2, SYCO: 2, SYLT: 1

16 0.501 7 0.547 VICR: 3, CIAR:4, TAOF: 1, RUSE: 1, SOCA: 4, SONE: 1,

SYNO: 1

17 0.798 6 0.489 MESA: 1, VICR: 4, VIRI: 2, CIAR: 1, TAOF: 1, ASSY: 1

18 0.807 4 0.356 VICR: 2, CIAR: 1, SOCA: 2, ASSY: 1

19 0.655 11 0.678 VICR: 4, CIAR: 1, TAOF: 1, EUGR: 3, SOCA: 3, SYNO: 2, SYPU: 1, MEPI: 2, MOFI: 3, NECA: 1, COSE: 1

20 0.817 5 0.463 VICR: 2, VIRI: 1, CIAR: 1, ASSY:1, COAR: 4

21 0.716 3 0.580 VICR: 3, SOCA: 3, SYER: 2

22 0.654 5 0.577 VICR: 4, DACA: 1, SOCA: 4, SYNO: 1, SYLT: 1, THOC: 1

23 0.663 4 0.616 VICR: 4, CIAR: 4, SOCA: 3, SYNO: 2

24 0.558 4 0.656 VICR: 2, TAOF: 1, SOCA: 4, SYNO: 1

25 0.685 4 0.743 VIRI: 1, CIAR: 3, SOCA: 2, ASSY: 2

26 0.502 10 0.525 VICR: 4, CIAR: 2, SOCA: 2, EUMA: 2, EUGR: 3, SOCA: 4,

SYNO: 4, SYLT: 1, SYPU: 2, MEPI: 3

27 0.740 6 0.550 MELU: 2, DACA: 4, RUSE: 2, ERPU: 1, COAR: 2, PLLA: 1

28 0.649 13 0.663 SILA: 1, POTR: 1, MEAL: 1, VICR: 1, OEBI: 1, VIRI: 2,

DACA: 1, TAOF: 3, RUSE: 4, SOCA: 4, SYNO: 3, SYLT: 1,

COAR: 1

29 0.602 5 0.561 VICR: 4, CIAR: 3, TAOF: 2, SOCA: 4, SYNO: 2

30 0.532 7 0.665 VICR: 3, CIAR: 3, RUSE: 1, SOCA: 4, SYNO: 3, SYLT: 1,

COAR: 1

31 0.797 6 0.627 MESA: 1, VICR: 4, CIAR: 4, SOCA: 1, ASSY: 1, LIVU: 3

32 0.692 17 0.427 VICR: 4, PONO: 2, RULE: 4, DACA: 2, CIAR: 2, TAOF: 1,

EUMA: 1, ACMI: 1, EUGR: 3, SOCA: 4, SYNO: 4, SYPU:1, MOFI: 1, MEAR: 1, CORU: 1, COSE: 1, LYCI: 1

33 0.806 13 0.486 RHTY: 1, MEAL: 1, VICR: 2, VIRI: 3, PAQU: 4, RUSE: 2, EUGR: 1, SOCA: 4, SYNO: 1, SYCO: 1, ERPU: 2, MOFI: 4

34 0.860 7 0.491 RHTY: 3, EUCY: 1, VICR: 3, SOCA: 4, COAR: 2, MOFI: 1,

LIVU: 3

35 0.550 10 0.637 VICR: 4, CIAR: 3, TAOF: 1, EUMA: 1, EUGR: 2, SOCA: 2, SYNO: 3, SYLT: 3, SYPU: 1, ASSY: 3

36 0.643 14 0.714 HYPE: 1, VICR: 1, DACA: 3, CIAR: 1, CIVU: 1, TAOF: 1,

EUGR: 2, SOCA: 4, SYNO: 2, VIRA: 4, ASSY: 3, PLLA: 1,

LIVU: 2, COSE: 1

37 0.756 7 0.439 VICR: 1, RULE: 4, CIAR: 3, SYNO: 2, SYLT: 4, ASSY: 4,

MEAR: 2

38 0.800 12 0.533 POTR: 2, MELU: 1, VICR: 4, OEBI: 1, VIRI: 1, PAQU: 2,

DACA: 4, RUSE: 1, LEVU: 1, SOCA: 4, ERPU: 2, PHHE: 2
